# Recent advances in colonoscopy

**DOI:** 10.12688/f1000research.18503.1

**Published:** 2019-07-09

**Authors:** Edward Seward

**Affiliations:** 1 Department of Gastrointestinal Services, University College London Hospitals NHS Trust, London, UK

**Keywords:** colonoscopy, colorectal cancer, polyps

## Abstract

Colonoscopy continues to evolve as equipment and techniques improve. Traditionally, colonoscopy has focused on adenoma detection, characterisation and resection as the primary aims, and there has certainly been considerable activity over the last few years in terms of addressing these important issues. This review article not only will discuss progress made in these areas but also will focus on when to colonoscope in terms of introduction of faecal immunochemical testing, how to insert with the advent of water-assisted insertion, and how to withdraw using a bundle of evidence-based techniques to improve adenoma detection. In addition, the ramifications of failing to discover polyps and of post-colonoscopy colorectal cancer are highlighted.

## Introduction

Colonoscopy continues to be the primary means of investigating lower gastrointestinal symptoms. Colonoscopy has been shown to reduce the risk of subsequent colorectal cancer both in screening ^1^ and in the symptomatic population ^2^. However, we do know that, despite its popularity, colonoscopy is not perfect, and post-colonoscopy cancer is a recognised feature. Therefore, it is worthwhile to ask the existential question, “What happens when colonoscopy goes wrong?” How to avoid this calamitous result will be discussed along with a consideration of the now-impressive data that exist for water-assisted insertion and how to optimise withdrawal as well as the prevention, management and follow-up of incomplete adenoma resection.

## Whom to colonoscope: the role of faecal immunochemical testing

Faecal immunochemical testing (FIT) has been used extensively in the screening population, where it outperforms traditional guaiac faecal occult blood (gFOB) testing in terms of both sensitivity for detecting pathology but also ease of use to improve screening participation ^3^. FIT differs from gFOB in that the former is specific to the globin portion of the haemoglobin molecule and therefore is specific to human haemoglobin, thereby reducing cross-reactivity with other mammalian forms of haemoglobin. It also provides a quantitative assessment of haemoglobin present in stool (measured in micrograms of haemoglobin per gram of stool) as opposed to the qualitative assessment in gFOB (as to whether or not a colour change has occurred). This allows a level of positivity to be chosen for a screening population (that is, a colonoscopy will be performed if the FIT level is more than 20 μg per gram of faeces); or in a symptomatic population, you may choose to make the test as sensitive as possible to decrease your chance of missing colorectal cancer (that is, any detectable faecal haemoglobin leads to a colonoscopy in the presence of colorectal symptoms). However, using FIT in the symptomatic population is challenging because although FIT has been shown to far outperform symptoms in terms of deciding who would benefit from a colonoscopy or computed tomography colonography ^4^, it can also be used to rule out the need for a colonoscopy and this is more controversial. A Scottish study ^5^ first highlighted the ability of FIT to be perfect (100% negative predictive value) in anticipating the absence of colorectal cancer if the FIT level was undetectable. If the FIT was undetectable in their population of both routine and urgent colorectal referrals (n = 755) who underwent both a FIT test and a colonoscopy, then no cancers were found, although about 1% had high-risk adenomas. Forty-two percent of the population had undetectable levels of faecal haemoglobin, meaning that if the decision of whether to undertake a colonoscopy had been based on the FIT result, this proportion would have been spared colonoscopy. This has obvious benefits for health systems where colonoscopy capacity acts as a constraint. It also improves convenience and safety for patients. However, subsequent publications have raised the possibility of “FIT-negative” cancer, which is colorectal cancer in the presence of undetectable levels of faecal haemoglobin ^6– 9^. Despite this risk, the negative predictive value for FIT is still probably over 99%, and the studies highlighted above suggest that FIT-negative cancers are the more clinically obvious ones (such as those accompanied by the presence of a mass, dramatic weight loss, and iron deficiency anaemia). Pathways incorporating FIT are being developed ^9^, but an essential component will be safety netting to ensure that potential cancers are not missed. Defenders of the use of FIT will point to the post-colonoscopy cancer rate associated with colonoscopy, which (as will be demonstrated) is not insignificant. Two further studies of over 12,000 patients in the UK are examining the use of FIT in symptomatic patients and results are due this year, so it may be that answers will be provided shortly.

## Colonoscopy insertion

Colonoscopy insertion is hampered by the discomfort that is caused by bowing and manipulation of the bowel by the colonoscope. This can be managed by using a magnetic imager that demonstrates the shape that the scope forms as it is inserted. Data on magnetic imagers have been available for over 20 years, but only recently did the largest meta-analysis to date come down in favour of their use for reducing time to caecum (even in expert hands), increasing caecal intubation rate and reducing pain scores ^10^. A more recent unblinded study ^11^ of propofol usage in a colonoscopy population was able to demonstrate increased satisfaction scores and reduced propofol use with a magnetic imager, and as a further study has demonstrated that propofol-supported colonoscopy is associated with higher complication rates ^12^, it is interesting to hypothesise that a suitably powered study could demonstrate improved safety as well. The most exciting contribution to improvements in colonoscopic insertion technique has been the advent of water-assisted insertion techniques. One of these is water immersion colonoscopy, in which water is used to assist insertion along with suction of air. In this way, the sigmoid loop is shortened and straightened. Another of these techniques is water exchange colonoscopy, in which water is both channelled in through the scope and sucked out at the same time, thereby cleaning the colon on insertion and maintaining a clear view on insertion. In both techniques, water is removed and carbon dioxide is insufflated on withdrawal in order to facilitate polyp identification and removal.

Data exist to support water-assisted colonoscopy and it has been the subject of a Cochrane Review that included 16 randomised controlled trials over a total of 2933 patients and demonstrated an increase in the adenoma rate and a reduction in pain and sedation requirements ^13^. A good review that describes the technique is available ^14^, and concerns about the additional time of water-assisted colonoscopy are addressed by a review and meta-analysis ^15^ that calculated that the procedure takes only two extra minutes for all the additional benefits for the patient.

## Colonoscopy withdrawal

Colonoscopic withdrawal, where polyps are identified and removed, is actually the most important phase of colonoscopy. Although significant attention has been placed on technological advances and the role that high-definition colonoscopes play in polyp detection, a clever quality improvement trial ^16^ demonstrated that a bundle of four very simple measures thought, when taken together, to improve adenoma detection (taking more than 6 minutes on withdrawal, retroflexing in the rectum, using buscopan routinely to abolish peristalsis, and turning on withdrawal to optimise exposure of the colon) could be taught as a package to endoscopists. The endoscopists in the study were divided into quartiles dependent on their pre-intervention adenoma detection rate (ADR) and assessed for their adenoma detection post-intervention. The study group found that ADR significantly improves in the worse-performing endoscopist quartile (an improvement from 7.3 to 14.9%), although better-performing quartiles failed to show significant improvement. This tells us that ADR can be improved in a poorly performing group by simple educational interventions. The same group ^17^ reported on the use of the Endocuff, a device that can be fitted on the end of a colonoscope and has multiple prongs to splay haustral folds on withdrawal, facilitating polyp detection. In that study, 1700 patients across seven hospitals took part, and adenoma detection increased significantly from 36.2 to 40.9%; the greatest benefit was in high-risk screening patients. Interestingly, the group also demonstrated a reduction in scope insertion time by 1 minute in the Endocuff group, theorising that it facilitates scope straightening. The rate of Endocuff removal, usually due to an angulated sigmoid, occurred in 4.1% because the Endocuff produced a small but appreciable effect on the colonoscope diameter.

Further recent data come from a very interesting article ^18^ comparing Endocuff with EndoRings (another end-of-the-scope device that again is designed to increase adenoma detection), full-spectrum endoscopy (a colonoscope with side viewing capability to produce a very-wide-angle view) and standard high-definition colonoscopy. Endocuff outperformed the other three modalities in terms of ADR and adenomas per colonoscopy (APCs), particularly in the right side of the colon. This was true for both high-detector colonoscopists and those with lower ADRs, averaging an improvement in adenoma detection across all groups of 7%. These data are encouraging but require tempering from a recent meta-analysis ^19^ of high-quality studies on the effect of Endocuff-assisted colonoscopy. Although the meta-analysis confirmed the benefit of Endocuff in adenoma detection (particularly amongst low to moderate detectors), the improvement was not as marked as previous studies suggested; the number needed to treat was 19, prompting considerations of cost. Therefore, in regard to detecting adenomas, colonoscopists need to be urged to do the basic things first ^20^ before reaching into their pockets and spending money on equipment to assist this. However the endoscopist does it, we know that there is a very strong inverse association between higher ADRs and a reduced risk of post-colonoscopy colorectal cancer (PCCRC) ^21, 22^ and so improving withdrawal technique and possibly using devices can only reduce the risk of this most feared complication.

A final point regarding adenoma detection on withdrawal is that it can be difficult to calculate and can be gamed ^23^. Focusing purely on ADR as a means of assessing the quality of a colonoscopist has been questioned as there is the anxiety that endoscopists will be tempted to relax their search for further adenomas once one has been discovered—the so-called “one and done” phenomenon ^24^—prompting calls to have alternative key performance indicators such as APCs or significant polyps detected per 6 minutes of withdrawal time ^25^. However, only ADR has been demonstrated to predict PCCRC rate, which is the “hardest” end point for quality of colonoscopy and therefore is still regarded as the gold standard.

## The reality of post-colonoscopy cancer

Every endoscopist knows the sinking feeling that occurs in a multi-disciplinary cancer meeting when the next patient up for discussion is revealed to have had an endoscopic procedure in the previous year. PCCRC is a recognised phenomenon but was not thoroughly described until the recent publication of a consensus statement from the World Endoscopy Organization ^26^. That article rigorously defined PCCRC as being between 6 and 36 months following the index colonoscopy; cases within less than 6 months were excluded to avoid counting cancers identified following a repeat colonoscopy (for inadequate bowel preparation, for instance). The article identified the PCCRC rate as an important metric of the performance of a *service* and not of an *individual* (as PCCRCs thankfully are relatively rare). A structure for evaluation of the PCCRC occurrence is suggested, and there are two basic questions: First, was there a previously resected polyp in the same segment as the cancer? In other words, could the resection have been inadequate? We already know from the CARE (Complete Adenoma Resection) study ^27^ that incomplete resection rates increase with size and sessile serrated morphology, such that almost half of large sessile serrated lesions were incompletely resected. Resection method may also be relevant, although a recent meta-analysis ^28^ found no difference between cold snare and hot snare resection in terms of polyp recurrence.

Second, was the quality of the colonoscopy adequate in regard to the extent and quality of bowel preparation? It is hoped that by defining the terms and providing a framework for prospective audit, we will gain a greater understanding of this difficult issue.

The scale of the problem is difficult to ascertain, resting as it does on the requirement for interrogation of national databases (as patients may have a colonoscopy in one hospital and have their cancer discovered in another). UK data suggest that the rate in the English National Health Service is 8.6% ^29^. The most recent data have been presented only in abstract form 30 but reassuringly demonstrate a fall to 7.5% from 2006 to 2012, presumably representing improvements in equipment, technique and bowel preparation. The authors made the point that variation between units demonstrated PCCRC rates of 5% in the highest-performing quintile and 11% in the lowest, which is clearly unacceptable. Falling PCCRC rates are not universal; a Canadian study ^31^ failed to demonstrate a reduction in PCCRC of about 8% between 1996 and 2010. Clearly, this represents a call to arms for all colonoscopists to adopt strategies to understand why PCCRCs occur, to employ techniques to improve adenoma detection, and to ensure complete resection as well as ensuring that adequate surveillance techniques are employed to manage the possibility of incomplete resection.

## Conclusions

Since the removal of the first polyp by endoscopy, colonoscopists have been striving to develop ways of improving their techniques. As the ultimate aim is to reduce the risk of colorectal cancer or even abolish it entirely, the PCCRC rate is a very useful metric, serving to inform at a service level how effective the unit is. Just how prospective audits will inform this process and whether we will see a reduction in PCCRC remain to be seen.

New methods of water-assisted insertion are available (with encouraging data to support their use) and are easy to learn. Future studies may focus on how it improves adenoma detection, be it through colon cleansing or the magnifying effect of water. The optimum technique and whether it may just be adequate to use water insertion in the left colon only have yet to be decided. End-of-the-scope devices such as Endocuff are exciting and seem to improve every clinician’s ability to spot polyps, although perhaps the benefit does not always justify the cost in every patient. However, the clinical significance of the discovery of additional multiple tiny adenomas is not clear and this too needs further evaluation.

Colonoscopy at its inception was regarded as a “quantum leap” over what was previously available. Recent iterations of improvement have demonstrated that this progress with colonoscopy continues and innovation remains at the heart of lower gastrointestinal endoscopy.

## Abbreviations

ADR, adenoma detection rate; APC, adenoma per colonoscopy; FIT, faecal immunochemical testing; gFOB, guaiac faecal occult blood; PCCRC, post-colonoscopy colorectal cancer
